# Influence of Parasitic Worm Infections on Allergy Diagnosis in Sub-Saharan Africa

**DOI:** 10.1007/s11882-017-0733-y

**Published:** 2017-09-01

**Authors:** Abena S. Amoah, Daniel A. Boakye, Maria Yazdanbakhsh, Ronald van Ree

**Affiliations:** 1 Department of Parasitology, Leiden University Medical Center, Albinusdreef 2, 2333 ZA Leiden, The Netherlands; 2 Department of Parasitology, Noguchi Memorial Institute for Medical Research, College of Health Sciences, University of Ghana, Accra, Ghana; 3 Department of Experimental Immunology, Academic Medical Center, Amsterdam, The Netherlands; 4 Department of Otorhinolaryngology, Academic Medical Center, Amsterdam, The Netherlands

**Keywords:** Allergy diagnosis, Allergic inflammation, Helminths, IgE cross-reactivity, Immune hyporesponsiveness, Sub-Saharan Africa

## Abstract

Epidemiological studies from Sub-Saharan Africa indicate that allergies are on the rise in this region especially in urban compared to rural areas. This increase has been linked to improved hygiene, lifestyle changes, and lower exposure to pathogens in childhood. Reduced exposure to parasitic worm (helminth) infections and allergy outcomes has been the focus of a number of population studies over the years. Paradoxically, there are parallels in the immune responses to helminths and to allergies. Both conditions are associated with elevated levels of immunoglobulin E, high numbers of T helper 2 cells, eosinophils, and mast cells. These immune parallels have meant that the diagnosis of allergies in parts of the world where helminths are endemic can be hampered. The aim of this review is to examine observations from population studies conducted in Sub-Saharan Africa that demonstrate how helminth infections influence the parameters used to diagnose allergy outcomes in this region. We explore specifically how helminth infections hinder the in vitro diagnosis of allergic sensitization, influence the clinical manifestations of allergy, and also the effect of anthelmintic treatment on allergy outcomes. Advancing our understanding of how helminths influence allergy diagnosis is imperative for the development of improved tools to assess, diagnose, and treat allergic disorders in both helminth-endemic and non-endemic countries worldwide.

## Introduction

Time trends indicate a sharp rise in the incidence of allergic disease worldwide especially among children [1]. This increase has also been seen in Sub-Saharan Africa [2, 3] where several population studies have observed a higher prevalence of allergic disease among urban compared to rural individuals [3–9]. Within these countries, improved hygiene, reduced pathogenic exposure during childhood, and the adoption of a so-called “western lifestyle” are all factors that explain this rise [10]. Of particular interest, in tropical areas of the world, is the relationship between exposure to parasitic worm (helminth) infections and allergic disease (Table 1).

**Table 1:** Studies linking helminth infections to allergic disease diagnosis in Sub-Saharan Africa

Mechanism	Helminth species	Observation	Reference
Helminth-induced IgE cross-reactivity and allergic sensitization diagnosis	Helminth species not specified	Elevated levels of IgE were observed to the oligosaccharide associated with delayed mammalian meat reactions, galactose-α-1,3-galactose (α-gal) among rural and urban Kenyans from areas where helminths were endemic. No direct link was made between α-gal sensitization and specific helminth species.	Commins et al. 2011 [11]
	Helminth species not specified	Raised levels of IgE to α-gal among Zimbabweans from a helminth-endemic rural area. Strong correlations were also observed between levels of IgE to α-gal and IgE to cat allergens Fel d 5 and cat dander extract. No direct link was made between α-gal and specific helminth species.	Arkestål et al. 2011 [12]
	*Ascaris lumbricoides*	Positive association found between specific IgE to *Ascaris* antigen and skin prick test reactivity to aeroallergens among urban adolescents in South Africa. Observed association could be explained by IgE cross-reactivity between *Ascaris* proteins and corresponding homologues in house dust mite and cockroach.	Levin et al. 2012 [13]
	*Schistosoma haematobium*	High levels of IgE to peanut extract among children in Ghana that did not translate into peanut skin prick test reactivity or peanut allergy symptoms. These levels were strongly associated with *S. haematobium* infection as well as correlated with IgE to cross-reactive carbohydrate determinants (CCDs).	Amoah et al. 2013 [14•]
Helminth-induced allergic inflammation	*Anisakis spp*	Bronchial hyper-reactivity and dermatitis in fish-processing workers in South Africa linked to exposure to *Anisakis spp.*	Nieuwenhuizen et al. 2006 [15]
	*A. lumbricoides*	Positive association observed between *A. lumbricoides* infection and airway hyper-responsiveness in urban and rural children in South Africa.	Calvert and Burney 2010 [7]
	*Onchocerca volvulus*	Pruritic skin disease simulating atopic dermatitis seen among Ethiopian immigrants in Israel. Individuals exhibiting skin condition found to be infected with *Onchocerca volvulus.*	Baum et al. 2014 [16•]
	*A. lumbricoides*	Positive association established between *A. lumbricoides* infection and reported wheeze in study participants greater than 5 years of age living in fishing communities around Lake Victoria in Uganda.	Webb et al. 2016 [17]
Effect of anthelmintic treatment on allergy outcome diagnosis	*A. lumbricoides, Trichuris trichiura S. haematobium*	Increased risk of skin prick test reactivity to house dust mite extract seen in Gabonese children following anthelmintic treatment for *S. haematobium* and intestinal helminths.	Van den Biggelaar 2004 [18]
	Hookworm *M. perstans* *S. mansoni* *Strongyloides stercoralis* *T. trichiura*	Treatment with albendazole for soil-transmitted helminths among pregnant women in Uganda was found to be strongly linked to a higher risk of doctor-diagnosed infantile eczema and reported recurrent wheeze in their offspring. Praziquantel treatment for *S. mansoni* in infected mothers was associated with a higher risk of infantile eczema but not among infants whose mothers were *S. mansoni* negative.	Mpairwe et al. 2011 [19•]
	Hookworm *S. mansoni*	Negative association between being positive for any helminth infection and allergy outcomes in Ethiopian immigrants in Israel. One year post-anthelmintic treatment, an increase in allergy symptoms was observed in treated individuals.	Stein et al. 2016 [20•]

Helminth infections remain highly prevalent in tropical areas with over 1.5 billion people worldwide thought to be chronically infected with at least one soil-transmitted helminth [21]. Moreover, an estimated 240 million individuals, mostly residing in Sub-Saharan Africa, are affected by the waterborne helminth disease schistosomiasis [22].

Despite the lack of geographical overlap in areas of the world with the highest burden of helminth infection and places most affected by allergic disease, strong parallels exist between these two conditions on the immunological level. Both are associated with the induction of strong T helper 2 (Th2) immune responses leading to elevated levels of immunoglobulin E (IgE) as well as the presence of eosinophils, mast cells, and Th2 cytokines [23, 24]. The immune parallels between these conditions have meant that the diagnosis of allergy outcomes in regions of the world where helminths are persistent can be hampered or result in unique clinical presentations [25••].

In this review, findings from population studies conducted in Sub-Saharan Africa that illustrate how helminth infections influence the parameters used to diagnose allergy outcomes are examined. We explore specifically how helminth infections hinder the in vitro diagnosis of allergic sensitization and influence the clinical manifestations of allergic disease.

## Diagnosis of Allergy Outcomes

The clinical diagnosis of IgE-mediated allergy in many countries is based primarily on a physical examination, history of symptoms, and the determination of allergic sensitization [26•]. Although less common in Sub-Saharan Africa, in many high income countries, food or drug challenges are additional tools routinely used in allergy diagnosis [26•].

Allergic sensitization—the production of serum-specific IgE against harmless antigens known as allergens—is a well-established factor in the development of allergic disease [27] and crucial to allergy diagnosis. The determination of allergic sensitization is based on the measurement of specific IgE to allergens as well as the assessment of skin reactivity to a panel of allergens.

In vitro assessment of allergic sensitization can be determined by the measurement of allergen-specific IgE by assays such as an enzyme-linked immunosorbent assay (ELISA), radioallergosorbent test (RAST), ImmunoCAP assay or more recently, microarray allergen assay [28].

Skin prick testing is used to assess allergic sensitization to a panel of environmental or food allergens as well as to negative and positive controls. A skin prick test (SPT) involves applying an allergen in liquid form to the skin of a patient, piercing the drop with a lancet and determining whether a reaction occurs within 15 to 20 min following application [29]. If the SPT is positive, mast cell degranulation will result in the formation of a wheal visible on the skin at the site of the lancet prick. A mean wheal diameter of ≥ 3 mm is generally accepted as a positive reaction and provides an indication of allergic sensitization [29]. The prick-to-prick test is a variation of the standard SPT in which a fresh allergen extract source such as a fruit or vegetable is used [30]. In high-income countries, a strong correlation is often observed between allergen-specific IgE and SPT reactivity while in less developed countries, the presence of specific IgE does not always translate into equivalent numbers of allergic skin reactions [10]. Over the years, evidence from population studies in Sub-Saharan Africa has shown how helminths influence the diagnosis of allergy from in vitro testing to clinical presentation.

## Helminth Infections and the Assessment of IgE Sensitization

In the late 1960s, S.G.O. Johansson and colleagues published one of the first studies linking the then newly characterized IgE antibody to helminth infections. Their investigation found that a group of pre-school children in Ethiopia had up to 20 times higher levels of total IgE compared to Swedish children and that a sub-group of Ethiopian children infected with the helminth *Ascaris lumbricoides* had 28 times higher levels of total IgE [31•]. Even in this early study, Johansson et al. postulated that helminth infection may be an important factor in stimulating the production of IgE in infected individuals [31•].

Subsequently, several investigations from Sub-Saharan Africa have observed raised levels of allergen-specific IgE related to helminth infection that did not translate into allergy symptoms [14•, 32, 33]. This phenomenon can be attributed to IgE cross-reactivity in which antibodies directed against one epitope recognize similar epitopes in homologous molecules [34, 35]. In the field of allergy, the allergen that induces the initial allergic reaction is termed the “primary sensitizer” and subsequent molecules are considered cross-reactive allergens [34].

Since the early 1980s, two forms of cross-reactivity linked to allergy have been described: cross-reactivity due to proteins and cross-reactivity due to the sugar structures on glycoproteins known as cross-reactive carbohydrate determinants (CCDs).

Cross-reactivity between plant-derived proteins is well-characterized especially in relation to food allergy [36]. Although less described, cross-reactivity between inverte-brate-derived allergens (such as from mite, cockroach, and shrimp) with helminth antigens is an emergent field of interest [37••, 38]. Several proteins found in helminths and other invertebrates might play a role in IgE cross-reactivity such as tropomyosin [35, 39–42], glutathione S-transferase (GST) [43, 44], and paramyosin [45]. However, a structural and immunological investigation has shown that cross-reactivity among homologous proteins, such as GSTs from *Ascaris* and cockroach or mite, is not always significant [46].

Although a number of population studies from South America have investigated IgE cross-reactivity between proteins from helminths such as *Ascaris lumbricoides* and homologues from house dust mite or cockroach, there are very few studies in this area from Sub-Saharan Africa. However, published literature provides indications that IgE cross-reactivity between homologous proteins may explain some observations made in studies from Africa over the years. For example, in urban black adolescents in Cape Town, South Africa, a positive association was seen between specific IgE to *Ascaris* antigen and SPT reactivity to aeroallergens (house dust mite and cockroach) [13]. Although not explored, the positive SPTs in that study could have been the result of IgE cross-reactivity between proteins from *Ascaris* such as tropomyosin or GST and their corresponding homologues in house dust mite and cockroach.

Aside from *Ascaris*, other helminths have been implicated in IgE cross-reactivity involving proteins. For example, cross-reactivity between the filarial nematode *Onchocerca volvulus* tropomyosin (OvTrop) and house dust mite tropomyosin (Der p 10) has been shown [40]. While this cross-reactivity may explain elevated levels of specific IgE to house dust mite seen in areas endemic for onchocerciasis (river blindness), it has yet to be demonstrated in population studies.

Cross-reactive carbohydrate determinants (CCDs) on insect and plant glycoproteins are carbohydrate components that have been linked to IgE cross-reactivity [47]. The most characterized N-glycan motifs associated with carbohydrate cross-reactivity are an α-(1,3)-linked fucose on a proximal *N*-acetylglucosamine and a β-(1,2)-linked xylose on a core mannose [48]. Aalberse and colleagues first reported IgE antibodies directed against CCDs in 1981 [49]. A subsequent study by the same group demonstrated that high levels of IgE against peanut extract in a Dutch cohort of grass pollen-allergic patients without symptoms of peanut allergy were due to IgE against CCDs [50].

The role of helminths in carbohydrate cross-reactivity was illustrated in an investigation in Ghana among 1604 school-children in which raised levels of IgE against whole peanut extract were observed that did not translate into peanut allergy symptoms [14•]. In that study, 18% of participants were peanut IgE-sensitized (Immunocap ≥ 0.35 kU/L), yet 92% were SPT negative [14•]. Current *S. haematobium* infection was also positively linked to peanut IgE sensitization as well as a strong correlation seen between IgE to CCDs and IgE to whole peanut extract. Inhibition assays in a subset of children showed that *S. haematobium* soluble egg antigen and the CCD marker bromelain were strong inhibitors of IgE binding to peanut extract [14•]. IgE to CCDs hampers in vitro testing of allergen-specific IgE through false positives and the blocking of IgE to CCDs has been demonstrated to improve the detection of allergen-specific IgE [51].

Although IgE to CCDs is generally thought to be biologically inactive, the clinical relevance of IgE to some CCDs has been reported in a study that found that about one third of CCD-positive sera from individuals with tomato allergy had biologically relevant CCD-specific IgE [52]. Moreover, IgE against the mammalian carbohydrate epitope galactose-α-1,3-galactose (α-gal) has been recently associated with two forms of anaphylaxis [53]. Firstly, anaphylactic reactions to the first infusion of the monoclonal antibody cetuximab among cancer patients undergoing therapy in the Southeastern USA [54] and the second being delayed onset reactions hours after the consumption of mammalian meat products [55]. These reactions have been seen in the Southeastern USA, Central America, Australia, and East Asia [53]. IgE directed against α-gal is thought to be induced by ticks and a strong correlation has been observed between reported history of tick bites and elevated levels of IgE to α-gal [56]. Although no direct link has been made between α-gal and helminths in Africa, raised levels of IgE to α-gal have also been seen in individuals from helminth-endemic areas in Kenya [11] and Zimbabwe [12]. More investigations are necessary to determine the source of α-gal sensitization in helminth-endemic areas in Sub-Saharan Africa and whether specific helminths play a role in this sensitization.

### Component-Resolved Diagnostics and Improving Allergy Diagnosis

IgE cross-reactivity between helminth antigens and environmental or food allergens demonstrates the limited diagnostic value of assessing IgE reactivity to whole allergen extracts in populations from helminth-endemic areas. Whole extracts are made up of multiple allergenic and non-allergenic components which often lack standardization in their allergen content [57]. In recent years, the field of in vitro allergy diagnostics has developed component resolved diagnostics (CRD) in which IgE sensitization to individual allergen molecules is assessed using purified natural and recombinant allergens [58]. Advances in biochemistry and molecular biology have facilitated the sequencing, synthesizing, and cloning of allergenic proteins which have all led to the production of recombinant allergens for CRD [59].

Among Ghanaian schoolchildren with elevated IgE to whole peanut extract that was strongly associated with *S. haematobium* infection, CRD in a subset showed that despite these high levels, very low responses to recombinant peanut allergens Ara h 1, 2, and 3 were seen [14]. Although not related to helminths, Wollmann and colleagues used CRD to investigate IgE reactivity to peanut allergen components (Ara h 1–3, 6, 8, 9) among allergic patients in Zimbabwe who were IgE-sensitized to whole peanut extract but had no symptoms of peanut allergy [60]. While 46% of these Zimbabwean patients had IgE to at least one of the highly allergenic peanut components, half of this patient cohort had elevated levels of IgE to CCDs [60]. Both these studies demonstrate how CRD can be a useful tool in improving the specificity of in vitro allergy diagnosis in Sub-Saharan Africa.

## Helminth Infections and Clinical Presentations of Allergic Disorders

### Helminth-Induced Allergic Inflammation

Research has shown that in some individuals who have low exposure to helminths such as travelers or recent migrants to a helminth-endemic area, robust inflammatory allergic responses can develop to helminths [61]. In addition, the life-cycles of particular helminths, such as *A. lumbricoides*, *Strongyloides stercoralis*, and hookworm, involve the migration of the larval stage through the lungs which may result in respiratory symptoms often associated with asthma such as wheeze and dyspnoea (shortness of breath) [62•].

Th2-mediated responses to migrating larvae during helminth infection provides one explanation for positive associations between *A. lumbricoides* infection and symptoms of asthma such as wheeze seen in a number of population studies in Sub-Saharan Africa. For example, in South Africa, an investigation found that *A. lumbricoides* infection among urban and rural children was associated with increased odds of a marker of airway hyperresponsiveness: exercise-induced bronchoconstriction [7]. More recently, a household survey of 2316 individuals conducted in fishing communities on Lake Victoria in Uganda, observed a positive association between *A. lumbricoides* infection, and reported wheeze among participants greater than 5 years of age [17]. It is important to note, however, that aside from migrating larval stages, cross-reactivity between helminth antigens such as tropomyosin or GST may also explain positive associations between helminth infection and symptoms of asthma as has been observed in Colombia [41].

Migrating larvae are also a feature of other helminth infections such as onchocerciasis where larvae known as microfilariae migrate to the skin, eyes, and organs [63]. Onchocercal skin disease is a clinical manifestation of immune responses to migrating microfilariae in the skin during onchocerciasis [63]. Two major forms of onchocercal skin disease exist: a mild form and a severe form [64]. The mild form of onchocercal skin disease is associated with chronic *Onchocerca volvulus* infection, and in this form, microfilariae induce a state of immune hyporesponsiveness in the host which ensures the survival of the parasite through the regulation of strong Th2 immune responses [64]. The more rare but severe hyperreactive form of onchocercal skin disease is characterized by severe skin inflammation and is associated with strong Th17 and Th2 inflammatory responses as well as reduced numbers of regulatory T cells [64]. In both forms of onchocercal skin disease, the death of microfilariae under the skin, either following chemotherapy or spontaneously, may induce a strong inflammatory reaction in the host and the release of mediators of the allergic immune response [61].

The effects of the reversal of immune hyporesponsiveness associated with chronic onchocerciasis have been illustrated by a small retrospective study of 27 Ethiopian immigrants in Israel who presented with pruritic skin disease simulating atopic dermatitis [16•]. In that study, half the patients with pruritic skin disease were tested for *Onchocerca* *volvus* and all those tested were found to be positive for onchocerciasis based on serology for the marker of active disease IgG4 or skin snip test [16•]. Interestingly, for 92% of these patients, symptoms of pruritic skin disease started on average 2 years following immigration to Israel. The authors noted that the majority of their patients displayed the hyperreactive form of onchocerciasis associated with low-level infections and strong Th2 responses [16•]. They also hypothesized that changes in exposure to environmental factors, resulting from migration from a developing country to a high-income country, may have triggered an immunological shift that played a role in the onset of symptoms of onchocerciasis [16•].

Aside from migrating larval stages, transient helminth infections where humans are not the definitive host like *Toxocara* spp*.* can also induce Th2 inflammatory responses in the airways or the skin [61]. This has been demonstrated in Sub-Saharan Africa by research on infections with the marine helminth *Anisakis*. Marine mammals are definitive hosts of *Anisakis* while fish and cephalopods (octopuses/squids/cuttlefish) are intermediate hosts for its larval stages [15]. Humans become accidental hosts of *Anisakis* by consumption of the larval stage of the parasite in undercooked/raw fish or cephalopods [65]. Symptoms of *Anisakis* infection can include gastro-allergic reactions as well as anaphylaxis while exposure through the respiratory tract or skin can result in anaphylaxis, rhinoconjunctivitis, asthma, and dermatitis [65]. Nieuwenhuizen et al. reported on bronchial hyperreactivity and dermatitis in fish-processing workers in South Africa as a result exposure to *Anisakis* [15]. Their investigation also included an experimental murine model which was used to demonstrate that the *Anisakis* infective larval stage (L3) induced strong Th2 immune responses in mice. A later study by the same group found that following epicutaneous exposure to *Anisakis* antigens in mice, the Th2 cytokine interleukin (IL)-13 played an important part in protein contact dermatitis while another Th2 cytokine IL-4 was key in driving systemic anaphylaxis [66].

There are no other published studies on *Anisakis* infection in humans in other parts of Sub-Saharan Africa. It is possible that this is as a result of under-reporting as many cases of the infection go undiagnosed [65].

### Effect of Anthelmintic Treatment on Allergy Outcome Diagnosis

Chronic helminth infections induce immune regulatory networks in the host which are characterized by regulatory T cells, regulatory B cells, and alternatively activated macrophages [67]. An anti-inflammatory environment is the end result and is typified by elevated levels of IL-10 and TGF-β as well as general T cell hyporesponsiveness and attenuation of responsiveness to so-called “bystander antigens” that include vaccines and allergens [67]. Although there have been inconsistencies in observations, a number of population studies from Sub-Saharan Africa have observed an inverse relationship between chronic helminth infections and allergy outcomes [33, 68–73]. Differences in helminth species, timing, and intensity of infection as well as host genetics all contribute to the variations observed in population studies [61, 74].

Removal of helminths by chemotherapy can reverse immune hyporesponsiveness to allergens associated with chronic helminth infections. Although observations have varied, several longitudinal studies conducted in helminth-endemic areas worldwide have observed a positive association between anthelmintic treatment and allergen skin reactivity [75–77]. However, some studies have also observed no effect of anthelmintic treatment [78], an effect on specific allergens only [79], or a temporal effect [80].

In Gabon, a randomized, controlled trial was conducted in which 317 children aged 5–13 years living in a helminth-endemic area were given anthelmintic treatment (a combination of praziquantel for *Schistosoma* infection and meben-dazole for intestinal helminths) or a placebo every 3 months and skin prick tested every 6 months for a 30 month follow-up period [18]. In that study, anthelmintic treatment was associated with increased risk of SPT reactivity to house dust mite extract (hazard ratio = 2.51; 95% CI, 1.85–3.41) and this increased risk was found to be mediated partly by a reduction in intestinal helminth infection (ascariasis and trichuriasis) [18].

The significance of early-life events and timing in the relationship between helminth infections and allergy outcomes was demonstrated by a large randomized, double-blind, placebo-controlled trial in Uganda. The trial established that treatment with albendazole (compared with placebo) for soil transmitted helminths among pregnant women was strongly associated with an increased risk of doctor-diagnosed infantile eczema in their children [19•]. Praziquantel treatment for *Schistosoma* infection in infected mothers was linked to a higher risk of doctor-diagnosed infantile eczema but had no effect among infants whose mothers were *S. mansoni* negative [19•]. The trial also found that albendazole treatment was positively correlated with reported recurrent wheeze [19•]. Subsequently, children from this birth cohort were randomized to receive quarterly doses of anthelmintic treatment (albendazole) from 15 months to 5 years [81]. By 5 years, albendazole treatment had no effect on atopic eczema but this is not unexpected given that helminth infection was low in this birth cohort [81].

Recently, Stein and colleagues examined the burden of helminth infections among 126 immigrants from Ethiopia newly arrived in Israel and related these to allergy outcomes [20•]. The most prevalent helminth infection detected was hookworm (73.1%) followed by *S. mansoni* infection (47.2%) [20•]. Among these newly arrived immigrants, a negative association was observed between any helminth infection and “allergy” which was defined as the concomitant presence of positive SPT to common environmental allergens and clinical symptoms allergy derived from a questionnaire [20•]. Less than half of the study participants consented to take anthelmintic treatment and 1 year later, a general increase in clinical allergy symptoms and SPT reactivity was seen [20•]. Both chemotherapy and the lack of exposure to helminths for re-infection are likely to have contributed to an increase in allergy outcomes. This study has implications for the diagnosis of clinical manifestations of allergy in both helminth-endemic and non-endemic areas worldwide.

## Future Perspectives

Studies from helminth-endemic areas in Sub-Saharan Africa on IgE cross-reactivity between helminth antigens and allergens have shown that the specificity and diagnostic accuracy of in vitro testing of allergy can be greatly improved by the use of recombinant allergens for the evaluation of IgE responses to allergens. Future investigations in this region could greatly benefit from the use of CRD.

Although allergy studies among Ethiopian immigrants in Israel have had small sample sizes, they provide pertinent examples of how changing environments due to intercontinental migration from a helminth-endemic region to a high income country can lead to clinical manifestations of allergy symptoms that are closely linked to helminth infections. With more global migration and movement of people, such observations have implications for improving allergy diagnosis not only in helminth-endemic areas but in other parts of the world.

Research from Sub-Saharan Africa has shown that novel allergens exist that are relevant to this region, particularly in relation to food allergy [82]. Additionally, the origins of sensitization to some allergens that have been well-characterized in other parts of the world remain unknown in Africa. For example, among Ghanaian schoolchildren with elevated IgE to peanut extract, high levels of biological active IgE to the recombinant peanut allergen Ara h 9 were seen in a few individuals [14•]. Ara h 9 is a part of the non-specific lipid transfer protein (LTP) allergen family and is known to be important in peanut allergy in the Mediterranean region [83]. The source of sensitization to LTPs in Ghana remains unknown and more studies are needed to investigate whether helminth infections play a role in inducing sensitization to this and other novel allergens.

Understanding the relationship between helminth infections and allergies has advanced our knowledge of how environmental factors that influence pathogenic exposures can hamper the diagnosis of allergy outcomes. Future investigations must build on our understanding of these links to generate new tools to diagnose, treat and prevent allergies not only in high income countries but also in the developing world where allergies are emerging as conditions of public health importance.
